# “My brain feels like a browser with 100 tabs open”: A longitudinal study of teachers’ mental health and well‐being during the COVID‐19 pandemic

**DOI:** 10.1111/bjep.12450

**Published:** 2021-08-01

**Authors:** Lisa E. Kim, Laura Oxley, Kathryn Asbury

**Affiliations:** ^1^ Department of Education University of York UK

**Keywords:** COVID‐19, teachers, mental health and well‐being, thematic analysis, longitudinal trajectory analysis

## Abstract

**Background:**

Teaching and caring for pupils during the COVID‐19 pandemic has been a challenge for many teachers, and its impact on teachers’ mental health and well‐being (MHWB) should be of great national and international concern.

**Aim and participants:**

This study examines 24 primary and secondary school teachers’ MHWB experiences across three time points (April, July, and November 2020) using longitudinal qualitative trajectory analysis.

**Method:**

We used a mixture of inductive and deductive coding, based on the Job Demands–Resources Model, to identify the job demands (aspects of the job that can be physically or psychologically costly) and job resources (aspects of the job that can buffer the effects of job demands and promote achievement and growth) teachers reported experiencing across the three time points.

**Results:**

Generally, teachers’ MHWB seemed to have declined throughout the pandemic, especially for primary school leaders. Six job demands contributed negatively to teachers’ MHWB (i.e., uncertainty, workload, negative perception of the profession, concern for others’ well‐being, health struggles, and multiple roles) and three job resources contributed positively to their MHWB (i.e., social support, work autonomy, and coping strategies).

**Conclusions:**

Policymakers and practitioners can support teachers’ MHWB by engaging in more collaborative communication and ensuring greater accessibility to sources of social support. These discussions and provisions will be crucial in supporting teachers, and thereby the educational system, both during and after the pandemic.

## Background

The COVID‐19 pandemic has negatively affected the mental health and well‐being (MHWB) of individuals worldwide (Holmes et al., 2020). Teachers, confronted with increased demands and limited resources since March 2020, have not been exempt from these effects (Kim & Asbury, 2020b). However, the dominant narrative around MHWB in schools has largely focused on students, and teachers’ experiences and needs have been less widely heard or considered (Lee, 2020). Understanding teachers’ MHWB is important in its own right, and because poor MHWB can have serious consequences for the profession. For example, it can lead to teachers leaving the profession (Madigan & Kim, 2021b), which can be financially costly for schools and the educational system (Carver‐Thomas & Darling‐Hammond, 2017) and detrimental to student outcomes (Madigan & Kim, 2021a). Many countries cannot afford these consequences, given widespread teacher shortages and high attrition rates (Schleicher, 2018). England, the focus of this study, is affected by falling retention levels since 2011 (Long & Danechi, 2021). Given the importance of teachers’ MHWB, particularly during the pandemic when they are under new forms of pressure, this longitudinal qualitative study examines the changes in the MHWB of primary and secondary school teachers across three time points in 2020.

### Changes over time

Various pandemic‐related events and changes throughout 2020 have affected teachers’ lives. In England, April was the time of lowest MHWB in the population, when the first national lockdown restrictions were imposed (see Public Health England, 2020 for a review). Moreover, teachers were regularly requested to implement and work within the bounds of last‐minute governmental decisions; including school closures for most pupils on 20 March 2020, schools reopenings for some year groups by mid‐June, schools reopenings for all pupils in September, and school closures for most pupils on 5 January 2021 (Department for Education, 2020b).

Teachers across the world needed to adapt quickly to continue delivery of education and to look after the welfare of the pupils in spite of the pandemic. As in many other countries, Senior Leadership Team members (SLTs; e.g., headteachers, deputy headteachers) and classroom teachers (CTs) in England experienced not only constant changes in their work, from which they recounted stories of joy and sense of achievement, but also sadness and frustration (Kim & Asbury, 2020b). For one, teachers needed to bear in mind the digital divide between their pupils, resulting from a lack of digital technology and/or reliable internet connection. The prevalence of this divide was highlighted by a report published two years ago, which showed that only 12% of 11–18 year olds in the United Kingdom had a laptop, desktop, or tablet to access the internet at home (Lloyds Bank, 2018). The UK government, in response, has provided 1.3 million laptops and tablets to disadvantaged pupils during the pandemic (Department for Education, 2020a). In this light, teachers responded in a variety of ways to continue to teach their pupils, ranging from sending paper workpacks home, to delivering almost fully online teaching (Bayrakdar & Guveli, 2020).

A sudden shift in the working environment while needing to maintain personal responsibility at home has taken a toll on the MHWB of many teachers all around the world (Kraft et al., 2020). Moreover, anxiety surrounding the pandemic and lack of administrative support seem to be important predictors of teacher burnout (Pressley, 2021), with many teachers reporting symptoms of stress, anxiety, and depression (Alves et al., 2021). These findings highlight the need to study teachers’ MHBW in this challenging period.

Demands on teachers, and the resources available to them, have fluctuated throughout the pandemic, which has had consequences for their MHWB. In England, a study examined the proportions of teachers self‐reporting scores of 8 and above on a 10‐point measure of work‐related anxiety between October 2019 and January 2021 (TeacherTapp, 2021). They found high peaks, marking high prevalence of anxiety, in March (a week before school lockdown), in May (when June school reopening announcements were made), and in January 2021 (announcement of third national lockdown and second partial school closures). Moreover, the peaks were higher for headteachers (e.g., 40% on 12 May 2020 and 54% on 5 January 2021) than CTs (e.g., 11% on 19 May and 28% on 5 January 2021). These figures, especially those for headteachers, are suggestive of increasing levels of pressure and a need to understand the experiences of teaching professionals, including senior leaders, in depth.

Given the shifting landscape of the pandemic, teachers' experiences have changed over time. Thus, a longitudinal examination of teachers’ experience throughout the pandemic is necessary. Qualitative approaches allow teachers to explain and clarify their subjective experiences, which can add nuance to our understanding of how teacher MHWB has been affected by the pandemic. A longitudinal qualitative study of teachers’ MHWB, focusing on both SLTs and CTs’ experiences, can enhance our understanding of the impact of COVID‐19 on the teaching profession and how we should move forward.

### Job demands and resources

According to the Job Demands–Resources Model (JD‐R model; Bakker & Demerouti, 2007), MHWB is affected by dual parallel processes: job demands (aspects of the job that can be physically or psychologically costly; e.g., workload, role conflict, performance evaluation) and job resources (aspects of the job that can buffer the effects of job demands and promote achievement and growth; e.g., social support and work autonomy). As a validated framework for studying teacher MHWB (see Viac & Fraser, 2020 for a review), we draw upon the JD‐R model to examine the range of job demands and job resources that teachers in England faced during the pandemic by November 2020.

Notable examples of job demands and job resources within this framework are workload and social support, respectively. Number of working hours is an indicator of workload and teachers in the United Kingdom are working a greater number of hours than their international counterparts (Allen, Benhenda, Jerrim, & Sims, 2020). One survey conducted during the pandemic found that 31% of teachers and 70% of SLTs reported working more than 51 hours/week on average, and identified high workload as one of the primary reasons they were considering leaving the profession (Education Support, 2020). Job resources can buffer the effects of job demands on MHWB (Bakker, Demerouti, & Euwema, 2005) and social support among colleagues can be beneficial in this regard (Hakanen, Bakker, & Schaufeli, 2006). However, at a time when many teachers have been forced to work from different locations (e.g., from their respective homes or from different parts of the school), teachers may have been appreciating this job resource even more, and providing and receiving social support in new and different ways. During this time of uncertainty in terms of how the pandemic will progress and how it will impact teachers’ work, understanding the job demands teachers are confronted with, and the job resources they are benefiting from, will be helpful as countries consider necessary preventive and intervention strategies to recover and to build back the educational system and the teaching workforce as they emerge from the pandemic.

### The current study

To our knowledge, this is the first study to draw upon longitudinal qualitative data to examine trajectories of teachers’ MHWB during COVID‐19. Specifically, we examined the job demands and job resources affecting teachers’ MHWB across three time points between April and November 2020 (RQ1) and asked whether there were differences in the experiences between SLTs and CTs (RQ2).

## Methods

### Participants, procedure, and measures

This study, which forms part of a larger project, comprises a sample of 71 interviews with 24 state school teachers in England (11 primary and 13 secondary; 6 male and 18 female) who had 1–32 years of teaching experience (*M* = 12.55, *SD* = 8.94). The participants (Ps) were grouped according to their school type (Primary or Secondary) and their teaching role (SLT or CT), resulting in four participant groups: Primary SLTs (Ps 1–5), Secondary SLTs (Ps 6–9), Primary CTs (Ps 10–15), and Secondary CTs (Ps 16–24).

To provide more context for the quotations we share, we have reported the gender of the teacher (Male/Female) as well as their teaching experience group akin to Gu and Day’s (2007) categories of Early Career Teacher (ECT; ≤5 years of experience), Mid‐Career Teacher (MCT; 6–18 years of experience), and Late Career Teacher (LCT; ≥19 years of experience).

Using a semi‐structured interview format, participants were interviewed by the same researchers (i.e., first and last author) over Zoom (2020) four times. Three of the time points form the content of the current study: 23 April–1 May 2020 (Time 1; about six weeks after the partial school closures); 13–17 July (Time 3; about two weeks before end of the academic year); and 2–11 November (Time 4; about nine weeks into the new academic year). As MHWB questions were not explicitly asked at Time 2, this time point was excluded from the current analysis. The participants were financially compensated for their time and the project received ethical approval from the researchers’ university department.

Participants responded freely to a series of open interview items regarding their MHWB, which were phrased to encourage them to explain and elaborate their answers. Specifically, at each time point, participants were asked to explain: ‘What impact has being a teacher during the coronavirus pandemic had on your mental health and wellbeing?’. At Times 3 and 4, the initial question was followed by two additional questions. The first question allowed participants to explain their current experience compared with the past: ‘You may remember that we previously asked you this same question in [month]. Would you say that your mental health and wellbeing has changed between then and now?’ The second question allowed them to elaborate their answer: ‘Why do you think there has been a change, or lack of change, over this time?’.

### Data analysis

The interviews were auto‐transcribed by Zoom and the transcriptions were anonymized, checked, and edited against the audio recording by the research team. One team member (i.e., second author) read and re‐read the data and generated initial codes and themes, which were assigned to the relevant sections of the transcript. The coding and its framework were modified, reapplied, and finalized after iterative discussions with the research team. Similarly, the same team member conducted the initial two sets of analyses, which were then modified, reapplied, and finalized after discussions with the research team. Disagreements in the codes and analyses were resolved by referring to past literature frameworks and practices, led by the first author.

At each time point, participants were asked whether they felt there had been changes in their MHWB and their responses were coded as positive change, negative change, or no change (i.e., pre‐pandemic vs. T1; T1 vs. T3; T3 vs. T4). Specifically, zero was used as a baseline to indicate MHWB prior to the pandemic and when individual participants described a positive change from one time point to the next they were given a score of +1, a negative change scored −1 and no change was scored as 0. A cumulative sum across the time points was calculated for each participant, resulting in a possible range between −3 and +3. For example, if a participant indicated a positive change at each time point, they would score +3 by T4. For each teacher group, an average of participants’ scores was calculated, from which a visual representation of MHWB trajectories for each group of participants was generated.

We then examined participants' responses to the rest of the questions regarding MHWB using reflexive thematic analysis (Braun & Clarke, 2013). Specifically, their responses were coded first inductively and then deductively in line with the JD‐R Model (Bakker & Demerouti, 2007), whereby codes were grouped into two themes: job demands (factors that participants indicated were detrimental to their MHWB) and job resources (factors that were supportive to their MHWB). Longitudinal trajectory analysis was then carried out, looking for common thematic patterns and changes between time points across each participant and their groups (Grossoehme & Lipstein, 2016).

## Results

We examined the changes in teachers’ MHWB across three time points in 2020 and specifically the job demands and job resources underlying these changes.

### Change over time

**Figure 1:**
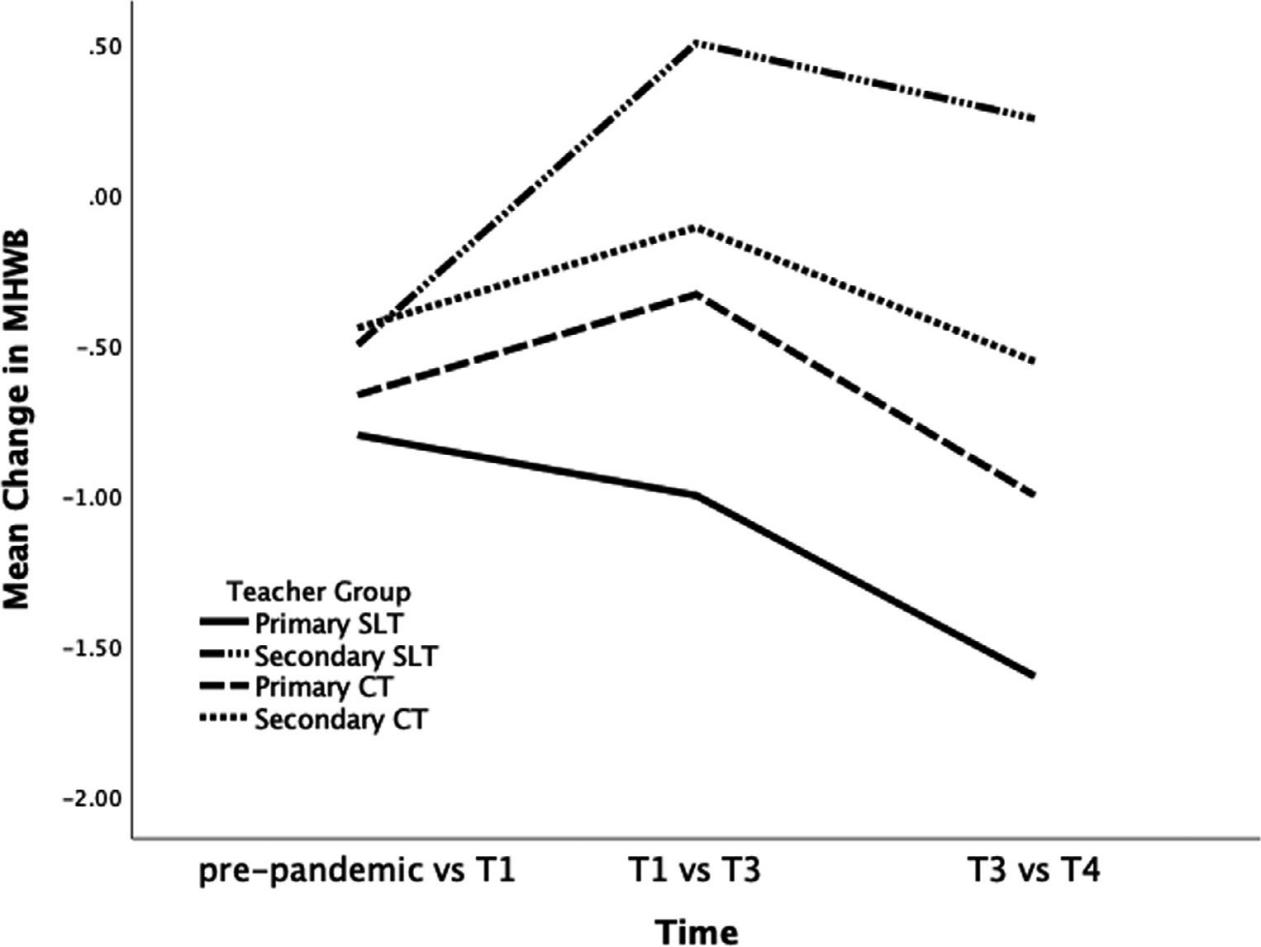
Changes in teachers’ mental health and well‐being between time points.

Figure 1 outlines the changes in MHWB across three time periods reported by participants across four teacher groups: Primary SLT, Primary CT, Secondary SLT, and Secondary CT. As can be seen, a negative impact was reported by all groups between T3 and T4. However, it is interesting to note that all groups, except for Primary SLT, reported a positive change in MHWB between T1 and T3. This finding may be attributed to a variety of factors, including the fact that T3 was a time with greater availability of social support because some year groups of pupils and teachers had returned to school, some national restrictions were lifted, and the end of the academic year was in sight. However, T3 was also the time when workload began to increase as teachers were required to teach those pupils who were in the classroom as well as those who were still at home. This suggests the possibility of a variety of job demands and resources that may have been at play in impacting their MHWB.

Secondary SLTs are an anomalous group as all participants in this group showed a significant positive change between T1 and T3 and, in contrast to the other groups, showed less of a negative overall impact. Participants in this group suggested that a positive change at T3 was due to experiencing less frustration and anxiety than they had at T1. They felt that the situation was calmer and that they were in a more stable routine, whereby ‘It's become more clear what we're actually doing and meant to be doing.’ (P8; Male Secondary SLT, T3).

Thus, we used an additional strategy to examine longitudinal changes in teachers’ MHWB. In line with the JD‐R model, two themes of job demands and job resources were identified in the data. Six job demands were identified as contributing to negative MHWB: uncertainty, workload, negative perception of the profession, concern for others’ well‐being, health struggles, and multiple roles. Three job resources were said to contribute to positive MHWB: social support, work autonomy, and coping strategies. A longitudinal matrix (see Table 1) outlines the changes in MHWB over time across the four teacher groups. Consistent with changes shown in Figure 1, the data suggests that the pandemic generally had a negative impact on the participants' MHWB though it was non‐linear. As Participant 15 put it, ‘I think it's been a rollercoaster. It's been up and down all over the place.’ (Female Secondary LCT, T3).

**Table 1:** Longitudinal analysis of mental health and well‐being across teacher groups

Themes	Codes	Teacher Group			
		Primary CT	Primary SLT	Secondary CT	Secondary SLT
Job demands	Uncertainty	Uncertainty was indicated across all time points.	Uncertainty over guidance created worry and was indicated across all time points.	Uncertainty was indicated across all time points.	Uncertainty was indicated across all time points
	Workload	As time went on, workload increased and changes to working practice created difficulties and led to increased stress.	Some SLTs felt that their workload did not increase over time, but just changed. Other SLTs indicated an increase in workload, with additional responsibility and quick decisions needed. Workload, while not necessarily increasing, was relentless and SLTs felt exhausted with the continuing situation.	As time went on, workload increased. Changes to working practice resulting in increased workload were highlighted at later Time points. Exhaustion highlighted at T3 and T4	A feeling of fatigue and exhaustion was highlighted and was more pronounced at T3 and T4.
	Negative perception of the profession	A lack of feeling valued as a teaching profession, due to social media and media portrayals of teachers, was mentioned at T4.	A lack of feeling valued as a teaching profession, due to social media and media portrayals of teachers, was mentioned across all time points.	A lack of feeling valued as a teaching profession, due to social media and media portrayals of teachers, was mentioned at T3 and 4. Confidence in government decisions was initially high, but this fell by T4, creating feelings of upset and anger.	A lack of feeling valued as a teaching profession was mentioned across all time points. For some, this led to questioning about whether to quit the profession at T1 and T3.
	Concern for others’ well‐being	Missing	Concern for the well‐being of other staff mentioned across all time points.	Concern for well‐being of other staff mentioned at T1 and T3.	Concern for well‐being of other staff mentioned across all time points.
	New and existing health struggles	Previous mental health struggles impacted on how well some participants felt they were coping. This was mentioned across all time points.	Previous mental health struggles impacted on how well some participants felt they were coping in the pandemic situation. This was mentioned at T3 and T4.	Physical health struggles were highlighted as being made worse by anxiety over the situation. Previous mental health struggles were highlighted as having an impact on how well participants felt they were coping. This was mentioned across all time points.	Missing
	Multiple roles	Competing pressures from SLT demands and parental expectations were highlighted at T3 and T4.	Missing	Missing	Missing
Job resources	Social support	Struggle with not having contact with others was more pronounced at T1, prior to the return to school. Contact with others (including friends and family as well as being back at school) was a protective factor at T3 and T4.	Support from colleagues and family was a protective factor across all time points.	Contact with others and support networks at home were highlighted as protective factors across all time points.	Contact with others and support networks in school were a protective factor across all time points.
	Work autonomy	At T1 and T3, some participants felt they had more time and autonomy. By T4, all participants felt they had less time. Having a routine was highlighted as a protective factor, particularly at T3.	Routine was highlighted as a protective factor at T4.	A sense of control was a protective factor indicated across all time points. Lack of control had a negative impact. Participants felt that they had more time at T1, with working from home being a protective factor. Routine also highlighted as a protective factor at T1.	Having a sense of control over the situation to some extent, by virtue of being SLT, was a protective factor, indicated at T1 and T3. Routine also highlighted as a protective factor at T1 and T3.
	Coping strategies	Missing	Existing coping strategies now being used in the new situation are mentioned across all time points.	Existing coping strategies mentioned at T1 and T3.	Existing coping strategies now being used in the new situation are mentioned across all time points.

### Job demands

Some types of job demands were more prevalent than others. For instance, uncertainty was mentioned by all teacher groups across all time points. However, multiple roles were raised as a concern only by Primary CTs. Job demands varied over time, and workload was particularly prominent at T3 and T4. The findings from this theme are presented in descending order starting from the factor that impacted on most teacher groups across the most time points.

#### Uncertainty

All teacher groups indicated uncertainty as a consistent detrimental influence on their MHWB across all time points. Participant 16, for instance, stated at the first time point, ‘I felt like and I still do feel like to a certain extent that I've had the rug ripped from under me. I feel like this is, I'm in a weird alternate universe where this is not quite happening.’ (Female Secondary ECT, T1). At a later time point, Participant 13 said, ‘The periods of uncertainty and not knowing and things constantly changing were very difficult in terms of just not knowing. I just find it very tiring to be honest and quite hard to process mentally.’ (Female Primary MCT, T3).

Uncertainty over government guidance to schools was indicated across all time points. Primary SLTs, such as Participant 4 particularly voiced their frustration ‘We're still working at this heightened level of managing people's anxieties and expectations and ever‐changing expectations about what we should be doing and how we shouldn't be doing it.’ (Female; T3). At T1, some Secondary CTs indicated sympathy with the government, despite the negative portrayal of teachers in the media. However, they expressed anger and frustration at government actions and decisions by T4. For instance, Participant 23 said, ‘Governmentally I am so cross with how they've dealt with it. Beyond cross.’ (Female Secondary LCT, T4). These frustrations stemmed from their lack of consultation with the education community and last‐minute decision making. The participants talked about these frustrations in the context of the negative impact they had had on their MHWB.

Uncertainty appeared to create heightened anxiety and meant that teachers were unable to plan their workload easily as they did not know when or how changes may occur. This was a detrimental factor for teachers’ MHWB and it was consistently talked about by participants across all the time points, indicating that the situation did not become less uncertain over time.

#### Workload

All teacher groups indicated that their workload increased over time, and that this had a negative impact on their MHWB. There was an initial feeling for some of having more time at the start of the pandemic. As Participant 11 stated, for instance: ‘I'm actually quite enjoying just having that time to look at the children's work online, having that time to catch up on paperwork and just relax a little bit.’ (Female Primary ECT, T1). However, at later time points, CTs at both primary and secondary schools indicated that ‘the workload is increasing exponentially at the moment.’ (P16; Female Secondary ECT, T4). Contributing factors included setting online work for pupils in addition to teaching in the classroom and additional cleaning routines that needed to be carried out between teaching classes. These factors made their jobs more difficult with little down time, which created extra pressure: ‘You've got this additional pressure of making sure everything's clean. Making sure that you're filling in those gaps.’ (P10; Female Primary ECT, T4). Moving between classrooms for different lessons, while ensuring adequate hygiene standards in line with government regulations, presented another additional pressure: ‘So for every lesson I have to move around to a different classroom, I have to set my stuff up, I have to antibac my hands, I have to set up. There's so much that you have to remember that it just absolutely owns your brain.’ (P21; Male Secondary MCT, T4).

Not all Primary SLTs seemed to perceive changes in their workload in the same way. Some Primary SLTs felt that their workload had not increased but that the nature of their work had changed. Participant 2 suggested that ‘it's not necessarily the amount of work because I think any teacher will tell you that there's always loads that you can be doing and you have to learn yourself when you switch off.’ (Female Primary SLT, T1). Additional responsibility and the need to make decisions quickly were all contributing factors to perceptions of an increased and different workload. Participant 1 said that ‘there are times when you just go, I can’t do this. Does that make sense? It’s just another thing on top of another thing or something else. You just think, what next?’ (Female Primary SLT, T3).

Even for those participants who felt that their workload had not necessarily increased, they indicated that the pace of their work was relentless. As Participant 16 put it, ‘I feel like I'm on overload. My brain feels like a browser with 100 tabs open. There is so much to think about all the time.’ (Female Secondary ECT, T4). Participant 20 talked about how difficult it was to keep up with the workload until the end of term: ‘That pace was really hard and I crawled towards half term. We're exhausted.’ (Female Secondary MCT, T4). The relentless nature of the work was described as leading to exhaustion in all teacher groups: ‘Exhausted. I'm emotionally exhausted. I'm physically exhausted … And I think to a certain extent, now I'm psychologically exhausted as well.’ (P4; Female Primary SLT, T3).

Increased and relentless workload appeared to have negatively affected teachers’ MHWB over time. The descriptions of feeling exhausted suggest possible early signs of burnout.

#### Negative perception of the profession

The feeling of being undervalued was mentioned across all time points. Participant 4, for instance, stated, ‘There's a lot of academics and a lot of other people saying schools need to stay open for the health and wellbeing of children. There's no conversation about staffing that, there's nothing about teachers.’ (Female Primary SLT, T4). Participant 9 worried about how others may be perceiving the profession while schools were closed for most pupils: ‘You've got the issue that people think you're at home on full pay doing nothing, which is not great for your mental health.’ (Male Secondary SLT, T1). For some participants, this issue led to questions about whether they wanted to continue in the teaching profession: ‘There were times when I felt, and feel, that I've had enough. I don't want to do this anymore, because you can't see any light at the end of the tunnel.’ (P8; Male Secondary SLT, T3).

Primary and Secondary CTs also mentioned the lack of feeling valued across later time points. The contrast between the increased workload teachers have been managing and the perception in the media that teachers were not working was highlighted by Participant 10: ‘I was working really hard and it almost feels like what we've been doing hasn't really meant anything.’ (Female Primary ECT, T4). Participant 17 described social media as being ‘toxic’ and said that ‘The teacher bashing on social media drives me absolutely mad. I've had to take myself off certain platforms because I can't read it anymore.’ (Female Secondary ECT, T4).

The negative perception of the teaching profession being portrayed in the media, and particularly on social media, had a detrimental impact on teachers’ MHWB. It left teachers feeling that their work was undervalued, and that the public did not have confidence in them as professionals. As Participant 10 put it, ‘It knocks your confidence a little bit and it just makes you feel like you're not doing anything that's purposeful.’ (Female Primary ECT, T4).

#### Concern for others’ well‐being

Primary and Secondary SLTs both raised concerns for the well‐being of other staff across all time points. Participant 6 expressed concern for staff managing additional workload: ‘Staff are running around and they're exhausted and I'm kind of concerned about their MHWB.’ (Female Secondary SLT, T4). SLTs indicated that they wanted to support other staff but were finding this difficult to balance with the other demands on their time. For example, Participant 1 expressed that: ‘I wish I could do more to help other people, but we all have our limits.’ (Female Primary SLT, T4). Responding to the need to be sensitive to staff MWHB meant additional pressures for SLTs, particularly primary SLTs. Participant 3, for instance, says that ‘It's definitely tougher than it's ever been before. I would say certainly this half term has been more stressful than I've ever known (it).’ (Female Primary SLT, T4).

Some Secondary CTs at T1 and T3 also voiced concerns for others, including Participant 20 who empathized with home schooling colleagues: ‘For somebody [a teacher] who has children and who is trying to teach as well as work, I could imagine that having quite a powerful impact on their wellbeing.’ (Female Secondary MCT, T1). Though CTs voiced their concern for others, it was more heavily voiced by SLTs.

#### Health struggles

This code encompasses a wide range of health factors. Several participants mentioned how previous health struggles had impacted on how well they felt they were coping in the current pandemic situation. Participant 15 indicated that the initial impact of the pandemic was difficult: ‘I've struggled with my mental health in the past anyway, so there have been moments when I've really struggled with it.’ (Female Primary LCT, T1).

Although previous ill health seems to have been described as a negative factor in relation to their current MHWB, some participants were also able to draw on coping strategies that they had employed in the past. Participant 22 described how they were able to support their own mental health by looking out for triggers:

I think as well because in the past I've struggled with mental health issues … I work very hard on my mental health and I know my triggers, and I know what I need to do. (Female Secondary MCT, T1)

New health conditions were also mentioned as being potentially caused by the ongoing pandemic situation and the uncertainty surrounding it. It was highlighted that this in turn negatively affected some participants’ MHWB. Participant 23, for instance, stated:

I think I've mentioned before that I had a stomach condition that arrived the day after lockdown, and it's still with me. I think it is literally my gut reaction to anxiety and stress. Outwardly I’m probably dealing with it, inwardly, not so much. (Female Secondary LCT, T4)

The impact of managing new and existing health conditions had a detrimental impact on teachers’ MHWB. However, there were also protective factors indicated by participants using coping strategies that had been successful in the past for maintaining their MHWB at this time.

#### Multiple roles

Over time Primary CTs increasingly raised the issue of competing demands on their time. By T3 and T4, there was a feeling that SLT instructions and parental expectations were not always aligned, leaving some teachers feeling confused and conflicted as to their duties and roles as teachers. Participant 11, for instance, felt that they were in a difficult position:

I feel like we're putting a bit of a burden on [parents] and their household, and the wellbeing in their household, which in turn we're feeling as teachers. But then we've got SLT they're saying to us, you need to do this, this and this. So we're feeling it from the parents, and we’re feeling it from SLT. (Female Primary ECT, T3)

This job demand appeared to increase over time, as it was not mentioned as being an issue at T1. This could be linked to the additional workload that teachers were being asked to manage at T3 and T4. There is also potentially a change in parental expectations and how well they are able to balance their own employment needs with supporting their child’s education. It is likely that parental stress increased over time and this was expressed to teachers in turn.

### Job resources

Some types of the job resources were more prevalent than others in supporting teachers’ MHWB. For instance, social support and work autonomy was mentioned by all teacher groups across all time points, whereas coping strategies were not mentioned by Primary CTs. As with job demands, job resources also varied over time. For instance, Primary CTs felt that they had increased work autonomy at T1 compared with at T3 and T4. The findings from this theme are presented in descending order starting from the factor that was said to affect most teacher groups across the most time points.

#### Social support

Contact with others was indicated to be a protective factor, which was mentioned by all teacher groups across all time points. Participant 18, for instance, talked about some of the positive relationships they had built during the pandemic: ‘I've just tried to make the best of those new relationships within the school and [I’m] trying to talk about anything but the current status quo. So that has helped a lot to build different friendships that I wouldn't have had.’ (Male Secondary ECT, T4). In addition to support from home and colleagues, some Primary CTs mentioned discussions on MHWB at school, ‘So they're very conscious about how well you’re doing.’ (P12; Female Primary ECT, T3).

Participants found the lack of social contact with colleagues and pupils difficult. This struggle was highlighted more strongly at T1, particularly by Primary CTs. For example, Participant 14 expressed that: ‘I'm struggling sometimes to be at home. Everything's just focused on the coronavirus. Not being at school to have different things going on as a distraction is really quite difficult.’ (Female Primary MCT, T1).

SLTs in particular highlighted a feeling of isolation across time points. Participant 7, for instance, expressed a ‘sense of sort of physical isolation, which definitely has an impact on your mental health’ (Female Secondary SLT, T4).

Opportunities for increased social contact with others, including friends and family as well as being back in school with colleagues and pupils, were a protective factor in teachers’ MHWB. Participant 15, for instance, talked about the importance of being able to keep in touch with family and friends: ‘I think because I've got those support networks in and, um, you know, I've got family and friends and stuff around that I can message and call and things I've managed to keep going through it.’ (Female Secondary LCT, T1).

There seemed to be a link between a positive change in MHWB and increased availability of social support when teachers returned to school in T3, though workload had increased. Thus, social support appears to be important for teachers’ MHWB.

#### Work autonomy

The sense of increased flexibility and being in control of their work and situation was found to be a protective factor. Some CTs initially felt that they had an increased amount of work autonomy. For example, Participant 19 said: ‘The more flexibility you've got in your day so you can go outside for half an hour, you can go for a walk for five minutes. That flexibility in being outside a lot helps.’ (Female Secondary MCT, T1).

Working from home and having a routine were identified as being protective factors for most teacher groups. For example, Participant 24 commented that ‘I think maybe you get a little more autonomy than you would in school…. there’s maybe a bit more autonomy about when you can choose to do things.’ (Male Secondary LCT, T3).

Feeling in control was said to have a positive impact on MHWB, whereas a lack of control was associated with a negative effect. Participant 23 expressed how difficult they had found the lack of control: ‘It's been tough. It's been really tough. The lack of control over the situation has been the main thing.’ (Female Secondary LCT, T4).

Secondary SLTs indicated that having control over the situation to some extent, as SLT were in a position to be making decisions, was a protective factor. Participant 6, for instance, said that:

Because of my role I've had quite a lot of autonomy and yes, you're having to respond to all sorts of government directions and you're having to respond to people who are superior to you, but in my own school community, being able to make your own decisions and your own choices, I think it's quite helpful actually. (Female Secondary SLT, T3)

Work autonomy appeared to impact the MHWB for all teacher groups, and the availability/use of this resource changed over time for CTs.

#### Coping strategies

All teacher groups, except for the Primary CT group, talked about new and existing coping strategies that they used to help them to maintain their MHWB during the pandemic, including exercise, DIY, and using a meditation app. Participant 7 talked about exercising and taking a break from meetings: ‘I'll do my meetings, I will exercise or walk or run or whatever, and then I come back to it.’ (Female Secondary SLT, T3). Participant 17 mentioned ‘using the Headspace [meditation app] as well… because I just needed to tap out of it [social media] and come away from constantly looking for negative things.’ (Female Secondary ECT, T3). These coping strategies were used across all time points. For example, the participant who was using the meditation app mentioned this across time points.

In sum, job resources seemed less plentiful than job demands, and social support appeared to be the strongest positive contributor to MHWB.

## Discussion

Throughout the pandemic, teachers have experienced increased job demands in the forms of uncertainty, workload, negative perception of the profession, concern for others’ well‐being, health struggles, and multiple roles. The impact of these job demands seemed to have been buffered by job resources, in the forms of social support, work autonomy, and coping strategies. However, the consequences of the potency of job demands outweighing that of job resources appeared to be experiences of stress and anxiety, exhaustion, and a lack of feeling valued as a profession. The general trend towards a worse MWHB from April to November 2020 seemed particularly marked for Primary SLTs.

### Job demands and job resources

Even before the pandemic, many teachers in England reported that the positive aspects of their profession do not triumph over its negative aspects, and that these negative aspects impact their MHWB (Ofsted, 2019). The balance between job demands and job resources seems to have continued to tip towards the former, and perhaps more so, throughout the pandemic.

Job demands that were recognizable in teachers’ lives before the pandemic (Viac & Fraser, 2020) were mentioned by our participants throughout the pandemic. Workload has been a persistent problem for teachers in England, indicated by working hours that are greater than their international counterparts and have not declined in the past 15 years (Allen et al., 2020). As our data indicates, workload has increased further for some teachers during 2020, which raises serious concerns about consequences such as burnout and thereby attrition (Madigan & Kim, 2021b), and negative impact on students (Madigan & Kim, 2021a).

A more nuanced factor of health struggles appeared in our data. This factor is worth highlighting in light of a quantitative longitudinal finding that women, young people (18–29 years), and those with pre‐existing mental health problems were particularly vulnerable to worse mental health throughout the first six weeks of lockdown in the United Kingdom (O’Connor et al., 2020). Teachers often fit in one or more of these categories, indicating possible greater vulnerability of the MHWB of certain teachers in the pandemic, especially when teaching can be stressful in itself. Health practitioners and policymakers, therefore, are encouraged to consider how certain populations within the teaching profession may need support from MHWB services, and how these groups can be most optimally supported at this time and beyond.

Teachers seemed to be making good use of job resources carried forward from pre‐pandemic times. Drawing on their existing social networks and coping strategies, such as social media channels and mindfulness exercises, was important for our teachers, which is consistent with practices of other teachers during the pandemic (Klapproth, Federkeil, Heinschke, & Jungmann, 2020). A greater sense of work autonomy—that is, ability to control what and when they do their work—was also a protective factor. Before the pandemic, allowing greater work autonomy as a vital pathway to support teacher MHWB was increasingly being highlighted as an important policy change needed at the school and national level (OECD, 2020). With a mindset shift that comes from a pandemic when norms are challenged, policymakers and practitioners are encouraged to seize this chance to start the process of considering policies that may further increase and support the sense of autonomy for teachers, which will in turn benefit teachers’ MHWB.

### Impact on SLTs and CTs

The MHWB of some members of SLTs, especially those in primary schools, seemed to have been negatively affected during the pandemic. Our findings are indeed in line with findings from other studies reporting general negative effects, especially for SLTs (e.g., Education Support, 2020; TeacherTapp, 2021). Greater impact on SLTs may be a reflection of their challenging responsibilities, including engaging in school planning and budgeting in the midst of the uncertainties of a pandemic, implementing last‐minute government guidance, and ensuring staff well‐being when they may be struggling themselves. Moreover, they may often be without the social support that CTs can often access and provide for each other due to concerns over perceived hierarchical boundaries (Hatcher, 2005) SLTs thus may be experiencing greater loneliness and emotional exhaustion during the pandemic.

Interestingly, our findings indicate that the reported experiences of Secondary SLTs differed from those of Primary SLTs. That is, secondary SLTs’ MHWB seemed to be not as negatively impacted by the pandemic as Primary SLTs. This may be the result of management structure differences between education levels, whereby secondary schools often have a greater number of leadership team members (e.g., middle management team members, such as heads of departments as well as senior management team members, such as deputy headteachers) among which workload can be shared out. This is in contrast to primary school management structures, which often consist of only one to three members (Earley et al., 2012). The effect of this factor is compounded by the usually greater interaction teachers have with parents in primary schools than secondary schools (Adams & Christenson, 2000), with which comes greater responsibility to communicate regularly with families. Moreover, Primary SLTs in England have had less time to implement necessary changes than Secondary SLTs. For example, when the government announcement was made for both primary and secondary schools to reopen in June for some year groups, primary schools were to open first followed by secondary schools a couple of weeks later. Thus, these findings may be a reflection of differences in distribution of the increased workload and the amount of time available to prepare and implement changes, raising concerns particularly for the MHWB of Primary SLTs.

### Practical implications

To benefit teachers’ MHWB, strategies can be implemented to decrease job demands and increase job resources. One way to decrease job demands— associated with workload, uncertainty, and lack of perception of feeling valued— is to ensure a collaborative and consultative communication line between government and the educational community when developing and implementing strategies during the pandemic and beyond (Kim & Asbury, 2020a; International Task Force on Teachers for Education, 2020). This will ensure that teachers can plan in advance of what may come in the future, and thus distribute their workload accordingly and also have reduced fear of uncertainties. A sense that teachers are being valued, as they contribute to the future of their profession in these governmental consultations and communications, should also benefit their MHWB. For example, this may be in the form of creating national and local communication channels through which representative members of the teaching community can work with independent bodies and/or policymakers to ensure practitioners’ perspectives are considered in national decision‐making.

Job resources can buffer the negative impacts of job demands on burnout (Bakker et al., 2005), thus attesting to the importance of ensuring teachers have sufficient resources that they can draw upon to prevent negative impact on their MHWB. For SLTs, ensuring they have reliable sources of social support, and that they feel comfortable accessing support, may be particularly important. Such sources of support may be in the forms of being mentored by a senior individual from outside of their school and participating in SLT forums dedicated to being a safe space to encourage and support each other. Future longitudinal quantitative studies may wish to examine the extent to which social support can buffer the negative impact job demands can have on the MHWB of teachers, including those of SLTs.

Schools are increasingly discussing how staff MHWB should be prioritized, in light of the pandemic and also the UK government's publication of the Education Staff Wellbeing Charter (Department for Education, 2021). Ensuring that schools have an established culture in which people feel safe and are confident that people care for each other, will be fundamental in continuing conversations on teacher MHWB (Kraft et al., 2020). Moreover, exploring how social support can be provided to teachers in a variety of formats — physical, virtual, and hybrid — may form one topic of this discussion that ensures that social support is provided in ways that flexibly accommodate the circumstances and needs of the teacher. Independent bodies may be helpful in providing some of this support, especially when local communities may not have the resources to do so.

### Limitations and future directions

Though these analyses were based on a sample of 71 interviews, it is not possible to generalize the study findings to the larger teaching population. The findings are reflective of our study participants' experiences and can only provide hints as to what other teachers’ experiences may be like. Accordingly, interpretations and generalizations should be approached with caution. Future studies with larger representative samples of the teaching profession would be helpful to capture a broader perspective on this topic.

Moreover, due to the pressures the participants were experiencing associated with the pandemic, the project aimed to ensure that being involved took up as little of their time as possible. Thus, member checking was not conducted to validate participants' responses prior to publication, though a pre‐published version of the paper was shared with the participants and met with a positive response. Nevertheless, future studies may wish to conduct member checking where possible.

Moreover, some participants found it difficult to reflect on how their MHWB changed over time as they felt they did not accurately recall their feelings at previous time points. Difficulty to recall past experiences is a common problem in retrospective studies (Dex, 1995). Future studies may benefit from reciting back to the participants what they had reported they had felt in the previous time points to aid their memory.

In line with Braun and Clarke’s (2013) practices and recommendations, the data coding and analysis was led by a single researcher, and the preliminary findings underwent iterative discussions as a research group. Some qualitative researchers establish inter‐rater reliability for their analyses, but this is not recommended for Braun and Clarke’s (2013) reflexive thematic analysis. Future studies, using a different analytic approach, may wish to do this as an additional way to reduce the possible subjectivity of the process.

The COVID‐19 pandemic has had a significant impact on teachers’ lives, with relentless job demands and limited job resources. Alleviating the sources of teachers’ strains and supporting teachers, where we can, is an important responsibility for all, now and as we emerge from the pandemic.

## Conflict of interest

All authors declare no conflict of interest.

## Author contributions

Kathryn Asbury (Conceptualization; Funding acquisition; Investigation; Writing – review & editing) Laura Oxley (Formal analysis; Methodology; Writing – original draft) Lisa E. Kim, Ph.D. (Conceptualization; Funding acquisition; Investigation; Methodology; Supervision; Writing – original draft; Writing – review & editing).

## Data availability statement

Data can be made available to researchers by contacting the corresponding author.
